# The risk of mild, moderate, and severe infections in inflammatory bowel disease patients—a prospective, multicenter observational cohort study

**DOI:** 10.1093/ecco-jcc/jjaf112

**Published:** 2025-07-09

**Authors:** Ashkan Rezazadeh Ardabili, Dirk van Esser, Dion Wintjens, Mia Cilissen, Debbie Deben, Zlatan Mujagic, Fritzi Russ, Laurents Stassen, Adriaan van Bodegraven, Dennis Wong, Bjorn Winkens, Daisy Jonkers, Mariëlle Romberg-Camps, Marieke Pierik

**Affiliations:** Department of Gastroenterology and Hepatology, Maastricht University Medical Centre+, Maastricht, The Netherlands; School for Nutrition and Translational Research in Metabolism (NUTRIM), Maastricht University Medical Centre+, Maastricht, The Netherlands; Department of Gastroenterology and Hepatology, Maastricht University Medical Centre+, Maastricht, The Netherlands; Department of Gastroenterology and Hepatology, Maastricht University Medical Centre+, Maastricht, The Netherlands; Department of Gastroenterology, Geriatrics, Internal and Intensive Care Medicine (Co-MIK), Zuyderland Medical Centre, Sittard-Geleen, The Netherlands; Department of Gastroenterology and Hepatology, Maastricht University Medical Centre+, Maastricht, The Netherlands; Department of Clinical Pharmacy, Clinical Pharmacology and Toxicology, Zuyderland Medical Centre, Sittard-Geleen, The Netherlands; Department of Gastroenterology and Hepatology, Maastricht University Medical Centre+, Maastricht, The Netherlands; School for Nutrition and Translational Research in Metabolism (NUTRIM), Maastricht University Medical Centre+, Maastricht, The Netherlands; Department of Gastroenterology, Geriatrics, Internal and Intensive Care Medicine (Co-MIK), Zuyderland Medical Centre, Sittard-Geleen, The Netherlands; School for Nutrition and Translational Research in Metabolism (NUTRIM), Maastricht University Medical Centre+, Maastricht, The Netherlands; Department of Surgery, Maastricht University Medical Centre+, Maastricht, The Netherlands; Department of Gastroenterology, Geriatrics, Internal and Intensive Care Medicine (Co-MIK), Zuyderland Medical Centre, Sittard-Geleen, The Netherlands; Department of Clinical Pharmacy, Clinical Pharmacology and Toxicology, Zuyderland Medical Centre, Sittard-Geleen, The Netherlands; Department of Methodology and Statistics, Care and Public Health Research Institute (CAPHRI), Maastricht University, Maastricht, The Netherlands; Department of Gastroenterology and Hepatology, Maastricht University Medical Centre+, Maastricht, The Netherlands; School for Nutrition and Translational Research in Metabolism (NUTRIM), Maastricht University Medical Centre+, Maastricht, The Netherlands; Department of Gastroenterology, Geriatrics, Internal and Intensive Care Medicine (Co-MIK), Zuyderland Medical Centre, Sittard-Geleen, The Netherlands; Department of Gastroenterology and Hepatology, Maastricht University Medical Centre+, Maastricht, The Netherlands; School for Nutrition and Translational Research in Metabolism (NUTRIM), Maastricht University Medical Centre+, Maastricht, The Netherlands

**Keywords:** remote monitoring tool, development, validation, infections, IBD, real-world data

## Abstract

**Background:**

In light of the growing number of treatment options, the benefit-risk balance of inflammatory bowel disease (IBD) drugs is increasingly important in clinical decision making. Postmarketing surveillance studies are pivotal to assess infection risk, yet mainly focus on severe infections. This study aimed to assess the incidence and risk factors associated with mild, moderate, and severe infections in IBD patients.

**Methods:**

We previously developed and validated a Patient-Reported Infections Questionnaire (PRIQ) which accurately assesses 15 infection categories with a 3-month recall period. The current prospective, multicenter, observational cohort study was performed between June 1, 2020 and July 1, 2021, enrolling consecutive IBD patients using myIBDcoach. Incidence rates (IRs) were calculated for all infections, and negative binomial regression was utilized to identify risk factors for infections over time.

**Results:**

In total, 629 IBD patients (*n* = 346 Crohn’s disease, *n* = 283 ulcerative colitis, 58.3% female) were included, completing 2397 PRIQs during 573.8 person-years (PY) of follow-up. This resulted in 991 reported infections and an overall IR of 172.7 per 100 PY, predominantly characterized by mild (IR 117.5 per 100 PY) and moderate (IR 50.9 per 100 PY) infections. Risk factors significantly associated with increased overall infection rates included female sex, higher comorbidity burden, smoking, and specific treatments, such as steroids, immunomodulators, anti-TNF agents, and JAK inhibitors, with steroids doubling infection risk (IR ratio 2.02).

**Conclusion:**

Mild and moderate infections are common among IBD patients and are particularly associated with both patient characteristics and specific immunosuppressive treatments. These findings emphasize the need for vigilant monitoring, especially for patients at higher infection risk, and allow for more personalized advice on benefit-risk of IBD treatments.

## 1. Introduction

The management of inflammatory bowel disease (IBD) has been revolutionized by the advent of immunomodulators and biological therapies. These therapies, although proven efficacious in managing disease activity, are associated with an increased risk of infections.^1–3^ Considering the rapidly growing number of treatment options, accurate assessment of the benefit-risk balance of these drugs is crucial in clinical decision making.

Traditionally, postmarketing surveillance studies and observational cohorts have predominantly focused on serious and opportunistic infections.^4–12^ This emphasis has created a considerable gap in our understanding of the true incidence of milder, albeit clinically relevant, infections among IBD patients. Similarly, data from randomized controlled trials (RCTs) often fall short in providing insights into these infections, as they typically exclude high-risk groups, such as elderly patients or those with multiple comorbidities. Although mild and moderate infections generally follow a benign course, they also impact work disability and quality of life, and can lead to prolonged recovery times in immunocompromised patients.^13^ Moreover, recurrent infections likely negatively affect therapy adherence, which may result in worse disease outcomes.^14–16^ These observations highlight the importance of integrating real-world data on mild and moderate infections into the benefit-risk assessment of treatment strategies in IBD.

So far, only a limited number of studies have specifically addressed milder infections. However, these studies were hampered by retrospective designs and the absence of validated measurement tools, resulting in bias and underestimation of true incidence of infection risks.^17–19^ Moreover, self-limiting infections and those commonly treated by general practitioners are not systematically captured in real-world settings, further complicating accurate assessment of their prevalence in IBD patients.

To address these methodological challenges, we previously developed and validated the Patient-Reported Infections Questionnaire (PRIQ), a 7-item remote monitoring tool covering 15 infection categories, which demonstrated excellent diagnostic accuracy to assess infections in real-world settings.^20^ In this multicenter prospective observational cohort study, leveraging the PRIQ, we aimed to assess the incidence and risk factors associated with mild, moderate, and severe infections in IBD patients.

## 2. Methods

### 2.1 Setting and study population

We conducted a prospective, multicenter, observational cohort study between June 1, 2020 and July 1, 2021 to evaluate the incidence of as well as identify risk factors for mild, moderate, and severe infections in patients with IBD using myIBDcoach.

MyIBDcoach is an established remote monitoring platform used for the management of IBD patients. Details on its development and clinical applications have been described elsewhere.^21^ In brief, myIBDcoach is an online telemedicine platform that tracks and periodically collects several patient-reported measures, including data on disease activity, side-effects, nutrition, lifestyle, and psychosocial factors. Whenever prespecified thresholds are exceeded, automated alerts notify the myIBDcoach back office, which is managed by nurse practitioners and IBD specialists. They can engage directly with patients and initiate intensified monitoring modules, e-learnings, and personal care plans, if deemed necessary. In the Netherlands, myIBDcoach is accepted as a standardized clinical care pathway with high patient satisfaction and platform adherence rates.^21^ Currently, myIBDcoach is integrated in the IBD care pathway of over 20 hospitals across the Netherlands and is actively used by more than 8000 IBD patients.

For the current study, all adult IBD patients who were actively using myIBDcoach and participated in the PRIQ development and validation study at Maastricht University Medical Center+ (MUMC+) and Zuyderland Medical Centre were eligible for inclusion.^20^ All study participants provided written informed consent. The study protocol received approval from the Ethics Committee of MUMC+ (MEC 2019-1115), adheres to the principles outlined in the revised version of the Declaration of Helsinki, follows the STROBE guidelines, and is registered at CilinicalTrials.gov (NCT04151420).^22^

### 2.2 Data collection

#### 2.2.1 Infection assessment

Details on the development and validation of the PRIQ have been described previously.^20^ In brief, the PRIQ is a 7-item questionnaire with a 3-month recall period that is designed to capture data on the types and subsequent treatment of infections. The PRIQ covers 15 infection categories, including flu or flu-like symptoms, respiratory tract (including COVID-19), urinary tract, skin (including warts and shingles), eye, genital, mouth, tuberculosis, meningitis, and hepatitis. Infection severity is determined by the type and route of treatment and is categorized into 3 severity levels: (1) mild: self-limiting or requiring topical antibiotics, antivirals, or antifungals; (2) moderate: requiring oral antibiotics, antifungals, or antivirals; and (3) severe: requiring hospitalization and/or IV antimicrobial treatment. The validation study demonstrated high diagnostic accuracy in a real-world setting.^20^ The PRIQ is implemented in myIBDcoach and is integrated into the periodic assessment of patients using the platform. During this current prospective cohort study, patients filled out the PRIQ in myIBDCoach during their 3-monthly periodic assessments.

#### 2.2.2 Covariates

Two types of covariates were considered. Time-fixed covariates were measured at the time of cohort entry and included age, sex, disease duration, IBD subtype, Montreal classification, smoking status, history of IBD surgery, comorbidities—including history of cardiovascular diseases, diabetes, kidney disease, neurological disease, liver disease, cancer, depression, psoriasis, primary sclerosing cholangitis, osteoarthritis, rheumatoid arthritis, and ankylosing spondylitis (including Charlson Comorbidity Index [CCI] based on comorbidities)—, body mass index (BMI), and history of serious infections. While the majority of these were extracted from myIBDcoach, all patient files were also manually reviewed by one of the authors (A.R.A.) for cross-validation and in order to extract covariates not systematically recorded in myIBDcoach, such as Montreal classification, comorbidities, and history and type of IBD surgery.

As for time-varying covariates, disease activity and medication exposure were considered. Given the importance of these covariates in relation to the measured outcome (ie, the risk of developing infections), their definitions and methods of assessment are discussed in more detail below.

##### 2.2.2.1 Disease activity

Disease activity was assessed at the time of cohort entry and subsequently updated at each periodic monitoring assessment in myIBDcoach. Disease activity was classified as either absent or present and was based on a composite assessment using the Monitor IBD at Home (MIAH) questionnaire, a validated patient-reported disease activity index, combined with fecal calprotectin (FCP) levels. Details on the MIAH are described elsewhere.^23^ In short, the MIAH is a validated patient-reported outcome measure (PROM) that can predict endoscopic inflammation. In its validation study, good diagnostic accuracy for predicting endoscopic activity was demonstrated when MIAH scores were combined with FCP levels.^23^ Given the observational nature of our study, routine standardized FCP assessments were not included in our study protocol. Therefore, FCP levels recorded within a 90-day window surrounding the periodic checks were considered. In the current study, disease was defined as active if patients had an MIAH score above the defined threshold and FCP levels >150 μg/g, and as inactive if both were below these thresholds. In instances where there was discrepancy between MIAH scores and FCP levels, the level of FCP ultimately determined the presence or absence of disease activity. In instances where no FCP result was available within the 90-day assessment window, disease activity classification was based on MIAH score alone.

##### 2.2.2.2 IBD medication exposure

In order to accurately assess IBD medication exposure during the 12-month prospective observational study period, a comprehensive analysis plan was implemented. Initially, prescription data for all patients were automatically extracted from hospital records. Subsequently, all electronic patient files were manually reviewed by one of the authors (A.R.A.) to ensure accuracy and completeness. During this process, all IBD medications, including their start and stop dates, were obtained and grouped by main mechanisms of action: immunomodulators (ie, thiopurines [azathioprine, 6-mercaptopurine, 6-thioguanine], methotrexate, cyclosporine, and tacrolimus), corticosteroids (ie, prednisone or budesonide), anti-TNF (ie, adalimumab, infliximab, certolizumab, and golimumab), anti-integrin (ie, vedolizumab and etrolizumab), anti-IL12/23 (ie, ustekinumab), JAK inhibitors (ie, tofacitinib, filgotinib, and upadacitinib), and S1P receptor agonists (ie, ozanimod and etrasimod), and modeled as time-varying covariates to reflect dynamic treatment patterns over time. Patients not using any medication or those receiving only oral or topical therapy with 5-aminosalicylic acid (5-ASA), given its non-immunosuppressive properties, served as the control group.

Medication exposure was assumed to begin at the reported start date. To ensure accurate assessment of exposure durations, washout periods for biologicals were defined based on the pharmacokinetics of the drugs by incorporating drug manufacturer data on half-life and input from IBD specialists, including pharmacists within our team. Washout periods were set at 30 days for all anti-TNFs, 75 days for vedolizumab, and 90 days for ustekinumab, corresponding to approximately 3 times the half-life of these drugs. For all other medications, exposure was considered to end on the recorded stop date. During follow-up, patients could be exposed to different medications simultaneously or successively, thereby potentially contributing person-time to multiple medication groups. All types of combination therapies were therefore considered in our analyses, but combination therapy with biologicals (especially anti-TNF) and immunomodulators were deemed of most clinical relevance.

### 2.3 Statistical analysis

Baseline characteristics are presented as means with corresponding SDs or median with first and third quartiles (Q1-Q3) for numerical variables, and as frequencies with percentages for categorical variables. If applicable, baseline data were compared for developing infection(s) (at least 1 vs no infection) using independent samples *t*-test for numerical variables and chi-squared test for categorical variables. The primary outcome of interest was the incidence of all (mild, moderate, and severe) infections as measured by the PRIQ. Secondary outcome of interest was the identification of risk factors for infections.

#### 2.3.1 Incidence of infections

The incidence of infections was assessed in terms of incidence rates (IRs) and calculated by dividing the number of infections by the sum of person-years (PY) at risk during the entire study period. PY at risk for each patient was defined as the time between study entry and the end of observation or loss to follow-up and was summed to obtain person-time at risk for the total study population. Crude IRs were calculated for all infections, stratified by infection severity (mild, moderate, and severe) and by both severity and infection site. IRs for overall infections and specific infection sites were expressed per 100 PY. Two-sided 95% CIs were calculated using the Mid-*P* exact test.^24^

#### 2.3.2 Risk factors for infections

In order to identify risk factors associated with the development of infections, we employed a 2-step approach. Initially, we conducted univariable negative binomial regression analyses to estimate the IR ratios (IRRs) for various predictor variables, including demographic characteristics and disease-related factors. Negative binomial regression is particularly suited for count data where the variance exceeds the mean, a common scenario in data involving infection counts, addressing the overdispersion that is inadequately modeled by Poisson regression.^25^

In the subsequent, more comprehensive phase, we adapted the Generalized Estimating Equation (GEE) framework to apply a negative binomial regression model that accommodates the longitudinal data structure, accounting for within-patient correlation and nonindependence of repeated measurements over time. In brief, GEE is an extension of generalized linear models that allows for the analysis of clustered or correlated data by modeling the correlations within groups of related observations, such as repeated measures from the same patients over time. Such a modeling approach is critical for analyzing data that involve time-varying covariates, such as changes in medication and disease activity. This approach allowed us to incorporate multiple variables simultaneously and is crucial for accurately capturing the dynamic nature of risk factors in a chronic disease setting where both exposure and outcome can vary over time. Treatment combinations were modeled as distinct categorical exposure categories. Treatment interaction terms were explored but were not included in the final model due to limited events in some strata and the risk of overfitting. Covariates included in the multivariable models were: age, sex, BMI, smoking status, CCI, disease activity, IBD subtype, and history of IBD surgery.

To ensure the robustness of our models, we rigorously tested several covariance structures, including first-order auto regressive (AR1), exchangeable, and unstructured. The unstructured covariance matrix was ultimately selected based on its superior fit, indicated by the lowest quasi-likelihood under the independence model criterion (QICc). We also examined potential issues of multicollinearity among predictor variables to validate stability and reliability of our models.

Outputs from the models are presented as IRRs, providing a relative measure of the effect of each predictor on the incidence of infections. Two-sided *P*-values ≤.05 were considered statistically significant. All analyses were performed using IBM SPSS for Windows (version 26.0, Armonk, NY), and plots were generated using R statistics (version 4.3.2) and RStudio (2024.01.04) using the ggplot2 package.

## 3. Results

### 3.1 Study population

A total of 629 IBD patients (347 [55.2%] Crohn’s disease [CD] and 282 [44.8%] ulcerative colitis [UC]) using myIBDcoach were included in the current prospective observational cohort study. As for distribution among centers, 223 patients (35.5%) were from MUMC+ and 406 (64.5%) from Zuyderland Medical Centre. Demographic and clinical baseline characteristics of all patients are presented in Table 1. Of these patients, 58.2% were females. Mean age and mean disease duration at time of cohort entry were 48.3 years (SD 14.8) and 12.4 years (SD 10.8), respectively. Regarding age at diagnosis, 58% of patients were diagnosed between 17 and 40 years (Montreal classification A2).

**Table 1.** Baseline characteristics of all IBD patients included in the prospective observational cohort study, and stratified by IBD type.

	All patients (*n* = 629)	CD (*n* = 347)	UC^a^ (*n* = 282)
Sex, *n* (%)			
Female	366 (58.2)	216 (62.4)	150 (53.2)
Male	263 (41.8)	131 (37.6)	132 (46.8)
Age at cohort entry in years, mean (SD)	48.3 (14.8)	46.0 (14.7)	51.1 (14.4)
Disease duration in years^b^, mean (SD)	12.4 (10.8)	12.6 (11.0)	12.2 (10.6)
Montreal A diagnosis^c^, *n* (%)			
A1: <17 y	30 (4.8)	21 (6.1)	9 (3.2)
A2: 17-40 y	365 (58.0)	218 (62.7)	147 (52.1)
A3: ≥40 y	234 (37.2)	108 (31.1)	126 (44.7)
Disease location^d^, *n* (%)			
E1: ulcerative proctitis	53 (18.8)		53 (18.8)
E2: left-sided	125 (44.3)		125 (44.3)
E3: extensive	104 (36.9)		104 (36.9)
L1: ileal	113 (32.6)	113 (32.6)	
L2: colonic	89 (25.6)	89 (25.6)	
L3: ileocolonic	145 (41.8)	145 (41.8)	
P: perianal involvement	75 (21.6)	75 (21.6)	
U: upper disease	16 (4.6)	16 (4.6)	
Disease behavior^d^, *n* (%)			
B1: inflammatory	261 (75.2)	261 (75.2)	
B2: stricturing	56 (16.1)	56 (16.1)	
B3: penetrating	30 (8.6)	30 (8.6)	
IBD surgery resection in history, *n* (%)	137 (21.6)	124 (35.7)	13 (4.6)
Stoma/pouch, *n* (%)	21 (3.3)	16 (4.6)	5 (1.8)
IBD in family^e^	184 (33.2)	104 (30.0)	80 (28.4)
Smoking status, *n* (%)			
Never	274 (43.6)	148 (42.7)	126 (44.7)
Ever smoked	281 (44.8)	137 (39.6)	144 (51.1)
Currently smoking	74 (11.8)	62 (17.9)	12 (4.3)
CCI, *n* (%)			
0	278 (44.2)	170 (49.0)	108 (38.3)
1-2	241 (38.3)	126 (36.3)	115 (40.8)
>2	110 (17.5)	51 (14.7)	59 (20.9)
BMI in kg/m^2^, mean (SD)	26.3 (4.7)	26.1 (4.8)	26.5 (4.6)
BMI category^f^, *n* (%)			
Underweight	11 (1.7)	8 (2.3)	3 (1.1)
Normal weight	270 (42.9)	155 (44.7)	115 (40.8)
Overweight	231 (36.7)	121 (34.9)	110 (39.0)
Obesity	117 (18.6)	63 (18.2)	54 (19.1)
History of serious infection, *n* (%)			
Tuberculosis	8 (1.3)	4 (1.2)	4 (1.5)
Hepatitis B	2 (0.3)	1 (0.3)	1 (0.4)
HIV	0 (0.0)	0 (0.0)	0 (0.0)
Baseline medication use^g^, *n* (%)			
No medication	213 (33.9)	87 (25.0)	126 (44.7)
IMM	88 (14.0)	52 (15.0)	36 (12.8)
Glucocorticoids	24 (3.8)	11 (3.2)	13 (4.6)
Anti-TNF	138 (21.9)	93 (26.8)	45 (16.0)
Anti-integrin	31 (4.9)	17 (4.9)	14 (5.0)
Anti-IL12/23	29 (4.6)	26 (7.5)	3 (1.0)
JAK inhibitors	6 (1.0)	3 (0.9)	3 (1.0)
Biological + IMM	53 (8.4)	32 (9.2)	21 (7.4)
Biological/IMM/SM + glucocorticoids	41 (6.5)	24 (6.9)	17 (6.0)
Biological + biological	6 (1.0)	2 (0.6)	4 (1.4)

Abbreviations: BMI, body mass index; CCI, Charlson Comorbidity Index; CD, Crohn’s disease; IBD, inflammatory bowel disease; IMM, immunomodulator; *n*, number of patients; UC, ulcerative colitis.

^a^UC including indeterminate colitis. In total 5 cases. ^b^Disease duration at time of cohort entry in years. ^c^Age at time of diagnosis according to Montreal classification. ^d^Current disease phenotype according to Montreal classification. ^e^IBD in family status not known in *n* = 57 (*n* = 34 CD, *n* = 38 UC) patients. ^f^Underweight < 18.50 kg/m^2^, normal weight 18.50-24.90 kg/m^2^, overweight 25.0-29.90 kg/m^2^, and obesity ≥ 30.0 kg/m^2^. ^g^Medication at time of cohort entry. No medication includes mesalamine; IMM includes thiopurines (azathioprine, 6-mercaptopurine, 6-thioguanine), tacrolimus, and ciclosporin; glucocorticoids includes prednisone and budesonide; anti-TNF includes infliximab, adalimumab, golimumab, and certolizumab; anti-integrin includes vedolizumab and etrolizumab; anti-IL12/23 includes ustekinumab; JAK inhibitors include tofacitinib, filgotinib, and upadacitinib. Small molecules (SM) include JAK inhibitors and S1P receptor agonists.

Of patients with CD, 41.8% had ileocolonic (L3) disease and 21.6% had a history of perianal disease involvement. Approximately one-third of CD patients had undergone at least one surgical resection at time of cohort entry. Among UC patients, disease was limited to the rectum (E1) in 18.8%, and 44.1% had left-sided colitis (E2). Further patient characteristics were similar between UC and CD patients, though notably, 14.7% of CD and 20.9% of UC patients had a CCI greater than 2.

As for medication use at time of cohort entry, 25% of CD patients were not on any maintenance medication, whereas nearly half (44.7%) of UC patients were using either no medication or 5-ASA monotherapy. About a quarter of CD patients were on anti-TNF monotherapy, and almost 10% of both CD and UC patients were on combination therapy with a biological and an immunomodulator. Other biologicals and small molecules were less commonly used (Table 1, Supplementary Table 1).

### 3.2 IR of infections

The 629 patients completed a total of 2397 periodic myIBDcoach checks (median 4.0 [Q1-Q3 3.0-5.0]), including the PRIQ assessments, during 573.8 PY of follow-up. Throughout this period of observation, a total of 991 infections were recorded, corresponding to an overall IR for infections of 172.7 per 100 PY (95% CI, 162.2-183.7). When stratifying for infection severity, mild infections were most commonly observed with an IR of 117.5 per 100 PY (95% CI, 108.8-126.6; *n* = 674 [68%]), followed by moderate infections at 50.9 per 100 PY (95% CI, 45.3-57.0; *n* = 292 [29.5%]), and severe infections at 4.4 per 100 PY (95% CI, 2.9-6.3; *n* = 25 [2.5%]) (Table 2).

**Table 2.** Incidence rates for all infections and stratified by infection severity per 100 PY.

	Events, *n*	PY at risk	IR per 100 PY [95% CI]
All infections	991	573.8	172.7 [162.2-183.7]
Mild infections	674	573.8	117.5 [108.8-126.6]
Moderate infections	292	573.8	50.9 [45.3-57.0]
Severe infections	25	573.8	4.4 [2.9-6.3]

Abbreviations: IR, incidence rate; *n*, number of infectious events; PY, person-years. 95% CI calculated using Mid-*P* exact test. Time at risk (573.8 PY) based on follow-up of 629 patients with inflammatory bowel disease during 1 y. PY at risk is slightly lower than the total amount of patients due do incomplete follow-up in a small proportion of patients.

Regarding specific infection categories, upper respiratory tract infections (URTIs) were most commonly observed with an IR of 35.7 per 100 PY (95% CI, 31.1-40.9), followed by influenza (IR 22.8 per 100 PY [95% CI, 19.2-27.0]), cold sores (IR 17.8 per 100 PY [95% CI, 14.6-21.6]), and urinary tract infections (UTIs) (IR 16.6 per 100 PY [95% CI, 13.5-20.2]). As for infection severity, for mild infections, URTIs were most reported (IR 26.9 per 100 PY [95% CI, 22.9-31.4]), whereas for moderate infections, UTIs were most common (IR 14.8 per 100 PY [95% CI, 12.0-18.3]). Severe infections were predominantly of gastrointestinal origin (IR 1.4 per 100 PY [95% CI, 0.6-2.6]). A sensitivity analysis excluding gastrointestinal infections that may have been flare-related (eg, intra-abdominal abscesses) reduced the overall IR for severe infections from 4.4 to 3.5 per 100 PY (95% CI, 2.2-5.3). Detailed IRs for each infection type and stratified by infection severity are listed in Table 3 and depicted in Figure 1.

**Table 3.** Incidence rates of infections per infection type and stratified by infection severity per 100 PY.

	All infections		Mild infections		Moderate infections		Severe infections	
Infection type^a^	*n*	IR [95% CI]	*n*	IR [95% CI]	*n*	IR [95% CI]	*n*	IR [95% CI]
Influenza	131	22.8 [19.2-27.0]	102	17.8 [14.6-21.5]	25	4.4 [2.9-6.4]	4	0.7 [0.2-1.7]
URTI	205	35.7[31.1-40.9]	154	26.9 [22.9-31.4]	48	8.4 [6.3-11.0]	3	0.5 [0.1-1.4]
LRTI	48	8.4[6.2-11.0]	14	2.4 [1.4-4.0]	31	5.4 [3.7-7.6]	3	0.5 [0.1-1.4]
COVID-19	46	8.0[5.9-10.6]	38	6.6 [4.8-9.0]	6	1.1 [0.4-2.2]	2	0.3 [0.06-1.2]
Eye	58	10.1[7.8-13.0]	53	9.3 [7.0-12.0]	5	0.9 [0.3-1.9]	0	N/A
Oral	76	13.2 [10.5-16.5]	58	10.1 [7.8-13.0]	17	3.0 [1.8-4.7]	1	0.2 [0.009-0.9]
Cold sore	102	17.8[14.6-21.6]	98	17.0 [14.0-20.8]	4	0.7 [0.2-1.7]	0	N/A
Gastrointestinal	18	3.1[1.9-4.9]	2	0.3 [0.06-1.2]	8	1.4 [0.6-2.6]	8	1.4 [0.6-2.6]
Urinary tract	95	16.6[13.5-20.2]	8	1.4 [0.6-2.7]	85	14.8 [12.0-18.3]	2	0.4[0.06-1.2]
Genital	33	5.8[4.0-8.0]	19	3.3 [2.0-5.1]	14	2.4 [1.4-4.0]	0	N/A
Skin	81	14.1 [11.3-17.5]	51	8.9[6.7-11.6]	30	5.2 [3.6-7.4]	0	N/A
Shingles	10	1.7[0.9-3.1]	1	0.2 [0.009-0.9]	9	1.6 [0.8-2.9]	0	N/A
Warts	68	11.9[9.3-15.0]	68	11.9 [9.3-15.0]	0	N/A	0	N/A
Tuberculosis	0	N/A	0	N/A	0	N/A	0	N/A
Meningitis	0	N/A	0	N/A	0	N/A	0	N/A
Hepatitis	3	0.5[0.1-1.4]	0	N/A	2	0.4 [0.06-1.2]	1	0.2 [0.009-0.9]
Other	17	3.0[1.8-4.7]	8	1.4 [0.6-2.6]	8	1.4 [0.6-2.7]	1	0.2 [0.009-0.9]
Totals	991	172.7[162.2-183.7]	674	117.5[108.8-126.6]	292	50.9[45.3-57.0]	25	4.4[2.9-6.3]

Abbreviations: IR, incidence rate; LRTI, lower respiratory tract infection; *n,* number of infectious events; PY, person-years; URTI, upper respiratory tract infection. 95% CI calculated using Mid-*P* exact test. ^a^Elaboration on infection types: URTI includes nasal cold, laryngitis, tonsillitis, sinus infection, ear infection, and “common” cold. LRTI includes pneumonia and bronchitis. Time at risk (583.8 PY) based on follow-up of 629 IBD patients during 1 y.

**Figure 1.**
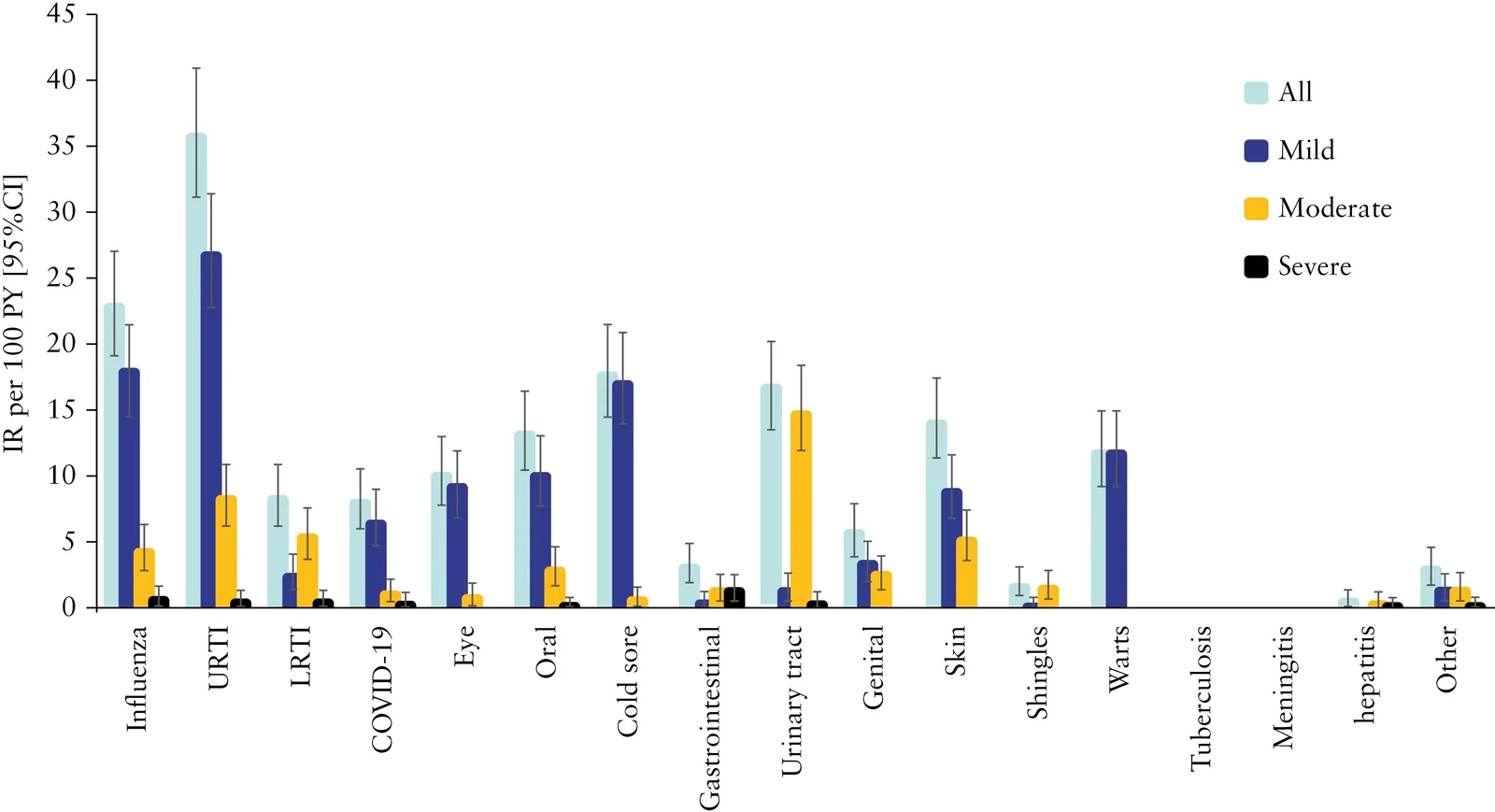
Incidence rates (IRs) of infections per infection type and stratified by infection severity per 100 person-years (PYs). Abbreviations: LRTI, lower respiratory tract infection; URTI, upper respiratory tract infection. 95% CI calculated using negative binomial regression. Elaboration on infection types: URTI includes nasal cold, laryngitis, tonsillitis, sinus infection, ear infection, and “common” cold. LRTI includes pneumonia and bronchitis.

### 3.3 Risk factors for infections

In examining predictors for development of infections, results from the univariable negative binomial regression analyses are presented in Table 4. Patients receiving any treatment had a statistically significant higher rate of all infections compared to patients without treatment, with an IRR of 1.49 (95% CI, 1.29-1.73, *P* < .001). This observation was consistent across mild infections (IRR 1.52 [95% CI, 1.27-1.81], *P* < .001) and moderate infections (IRR 1.45 [95% CI, 1.11-1.91], *P* = .004).

**Table 4.** Stratified incidence rates by infection severity and corresponding incidence rate ratio.

	*N*	PY at risk	Events	IR 100 PY [95% CI]	IRR [95% CI]	*P*-value
All infections						
No treatment	213	196.1	256	130.5 [115.3-147.3]	Ref.	
Treatment	416	377.7	735	194.6 [180.9-209.1]	**1.49 [1.29-1.73]**	**<.001**
Male	263	249.9	337	134.9 [121.0-149.8]	Ref.	
Female	366	323.9	654	201.9 [186.9-217.8]	**1.50 [1.31-1.71]**	**<.001**
Ulcerative colitis	282	257.5	409	158.8 [144.0-174.8]	Ref.	
Crohn’s disease	347	316.3	582	184.0 [169.5-199.4]	**1.16 [1.02-1.31]**	**.02**
<55 y	392	346.3	574	165.8 [152.6-179.7]	Ref.	
≥55 y	237	227.5	417	183.3 [166.3-201.5]	1.11 [0.97-1.25]	.12
CCI 0-2	519	466.4	714	153.1 [142.2-164.6]	Ref.	
CCI > 2	110	107.4	277	257.9 [228.9-289.7]	**1.69 [1.47-1.94]**	**<.001**
Mild infections						
No treatment	213	196.1	172	87.7 [75.3-101.6]	Ref.	
Treatment	416	377.7	502	132.9 [121.7-144.9]	**1.52 [1.27-1.81]**	**<.001**
Male	263	249.9	235	94.0 [82.6-106.7]	Ref.	
Female	366	323.9	439	135.5 [123.3-148.7]	**1.44 [1.23-1.69]**	**<.001**
Ulcerative colitis	282	257.5	288	111.8 [99.5-125.3]	Ref.	
Crohn’s disease	347	316.3	386	122.0 [110.3-134.7]	1.09 [0.94-1.27]	.26
<55 y	392	346.3	396	114.4 [103.5-126.0]	Ref.	
≥55 y	237	227.5	278	122.2 [108.5-137.2]	1.07 [0.92-1.25]	.40
CCI 0-2	519	466.4	492	105.5 [96.5-115.1]	Ref.	
CCI > 2	110	107.4	182	169.5 [146.1-195.5]	**1.61 [1.36-1.90]**	**<.001**
Moderate infections						
No treatment	213	196.1	77	39.3 [31.2-48.8]	Ref.	
Treatment	416	377.7	215	56.9 [49.7-64.9]	**1.45 [1.11-1.91]**	**.004**
Male	263	249.9	98	39.2 [32.0-47.6]	Ref.	
Female	366	323.9	194	59.9 [51.9-68.8]	**1.53 [1.20-1.95]**	**<.001**
Ulcerative colitis	282	257.5	106	41.2 [33.9-49.6]	Ref.	
Crohn’s disease	347	316.3	186	58.8 [50.8-67.7]	**1.43 [1.13-1.81]**	**.003**
<55 y	392	346.3	162	46.8 [40.0-54.4]	Ref.	
≥55 y	237	227.5	130	57.1 [47.9-67.6]	1.22 [0.97-1.54]	.09
CCI 0-2	519	466.4	203	43.5 [37.8-49.8]	Ref.	
CCI > 2	110	107.4	89	82.9 [67.0-101.5]	**1.90 [1.48-2.44]**	**<.001**
Severe infections						
No treatment	213	196.1	7	3.6 [1.6-7.1]	Ref.	
Treatment	416	377.7	18	4.8 [2.9-7.4]	1.34 [0.53-3.78]	.52
Male	263	249.9	4	1.6 [0.5-3.9]	Ref.	
Female	366	323.9	21	6.5 [4.1-9.7]	**4.05 [1.39-11.80]**	**.01**
Ulcerative colitis	282	257.5	15	5.8 [3.4-9.4]	Ref.	
Crohn’s disease	347	316.3	10	3.2 [1.6-5.6]	0.54 [0.24-1.21]	.13
<55 y	393	346.3	16	4.6 [2.7-7.3]	Ref.	
≥55 y	237	227.5	9	4.0 [1.9-7.3]	0.86 [0.38-1.94]	.71
CCI 0-2	519	466.4	19	4.1 [2.5-6.2]	Ref.	
CCI > 2	110	107.4	6	5.6 [2.3-11.6]	1.37 [0.55-3.43]	.49

Abbreviations: IR, incidence rate; IRR, incidence rate ratio; *N*, number of patients; PY, person-years; Ref., reference category. IRR and corresponding 95% CIs were calculated using negative binomial regression analysis. IRR displayed in bold are statistically significant.

As for demographic factors, female patients had a higher risk of infections compared to male patients across all infection severities, with an overall IRR of 1.50 (95% CI, 1.31-1.71, *P* < .001). Furthermore, IBD patients with a CCI greater than 2 had statistically significant higher rates of infections compared to patients with CCIs between 0 and 2, with IRRs of 1.69 (95% CI, 1.47-1.94, *P* < .001) for all infections, 1.61 (95% CI, 1.36-1.90, *P* < .001) for mild infections, and 1.90 (95% CI, 1.48-2.44, *P* < .001) for moderate infections. On sensitivity analysis excluding UTIs, a condition known to primarily affect females (82 out of 95 UTI [86.3%] cases in our study), the association between female sex and higher infection rates persisted compared to males (IRR 1.36 [95% CI, 1.19-1.56], *P* < .001).

Expanding on these initial findings, the subsequent multivariable negative binomial regression analysis assessed the simultaneous effect of various demographic, disease-, and treatment-related factors on infection rates. Results are presented in Table 5. Compared to patients without treatment, immunomodulator monotherapy (IRR 1.47 [95% CI, 1.05-2.05], *P* = .025), anti-TNF monotherapy (IRR 1.41 [95% CI, 1.06-1.88], *P* = .018), and combination therapy with anti-TNF and immunomodulators (IRR 1.65 [95% CI, 1.05-2.59], *P* = .029) were all associated with a statistically significant increase in rate of any infections. JAK inhibitor use (IRR 1.89 [95% CI, 1.10-3.25], *P* = .021) was also associated with higher rates of infections, and patients on glucocorticoids (IRR 2.02 [95% CI, 1.06-3.84], *P* = .033) showed more than a doubling in rates of infections. Anti-integrin and anti-IL12/23 monotherapy were not significantly associated with higher rates of infection compared to no treatment (Figure 2). Disease activity was not independently associated with increased infection rates (IRR 0.98 [95% CI, 0.79-1.20], *P* = .82). In a sensitivity analysis prioritizing clinical symptoms (MIAH score) over FCP in cases of discrepancy, disease activity was independently associated with increased rates of infections (IRR 1.30 [95% CI, 1.07-1.59], *P* = .009), while all other statistically significant associations remained unchanged (Supplementary Table 3).

**Table 5.** Incidence rate ratios for associations between predictors and subsequent risk of development of infection in patients with IBD using negative binomial regression.

	All infections	
	IRR [95% CI]	*P-*value
Medication groups		
No medication	Ref.	
IMM	**1.47 [1.05-2.05]**	.025*
Glucocorticoids	**2.02 [1.06-3.84]**	.033*
Anti-TNF	**1.41 [1.06-1.88]**	.018*
Anti-integrin	1.40 [0.85-2.29]	.19
Anti-IL12/23	1.09 [0.70-1.69]	.71
JAK inhibitors	**1.89 [1.10-3.25]**	.021*
Anti-TNF + IMM	**1.65 [1.05-2.59]**	.029*
Anti-integrin + IMM	1.54 [0.51-4.66]	.44
Anti-IL12/23 + IMM	1.20 [0.65-2.20]	.57
IMM/biological/JAKi + glucocorticoids	0.98 [0.65-1.49]	.92
Biological + biological	0.85 [0.47-1.52]	.59
Age at cohort entry	1.00 [0.98-1.02]	.83
Sex, female	**1.70 [1.35-2.14]**	<.001*
Disease activity, yes	0.98 [0.79-1.20]	.82
Disease entity, UC	0.95 [0.75-1.21]	.67
Smoking		
Never	Ref.	
Former	1.36 [0.94-1.96]	.11
Current	**1.43 [1.12-1.81]**	.004*
Charlson Comorbidity Index		
0	Ref.	
1-2	0.99 [0.70-1.42]	.45
>2	**1.69 [1.05-2.74]**	.031*
BMI category		
Normal weight	Ref.	
Underweight	1.17 [0.40-3.38]	.78
Overweight	1.06 [0.83-1.34]	.66
Obesity	1.28 [0.95-1.73]	.11
IBD surgery resection history, yes	1.04 [0.77-1.39]	.82

Abbreviations: BMI, body mass index; IBD, inflammatory bowel disease; IMM, immunomodulator; IRR, incidence rate ratio; Ref., reference category; UC, ulcerative colitis. An IRR greater than 1 represents an increase in the risk of developing an infection based on the absence/presence (categorical) or increase in 1 unit value (continuous) of an predictor. Generalized Estimating Equation (GEE) model includes both time-varying predictors (ie, medication groups, disease activity) and time-invariant predictors (ie, value at cohort entry: age, sex, disease entity, smoking status, Charlson Comorbidity Index, BMI, and IBD surgery history). No medication includes mesalamine; IMM includes thiopurines (azathioprine, 6-mercaptopurine, 6-thioguanine), tacrolimus, and ciclosporin; glucocorticoids includes prednisone and budesonide; anti-TNF includes infliximab, adalimumab, golimumab, and certolizumab; anti-integrin includes vedolizumab and etrolizumab; anti-IL12/23 includes ustekinumab; JAK inhibitors include tofacitinib, filgotinib, and upadacitinib. BMI: underweight < 18.50 kg/m^2^, normal weight 18.50-24.90 kg/m^2^, overweight 25.0-29.90 kg/m^2^, and obesity ≥ 30.0 kg/m^2^.

*Significant *P* value <.05. IRR displayed in bold are statistically significant.

**Figure 2.**
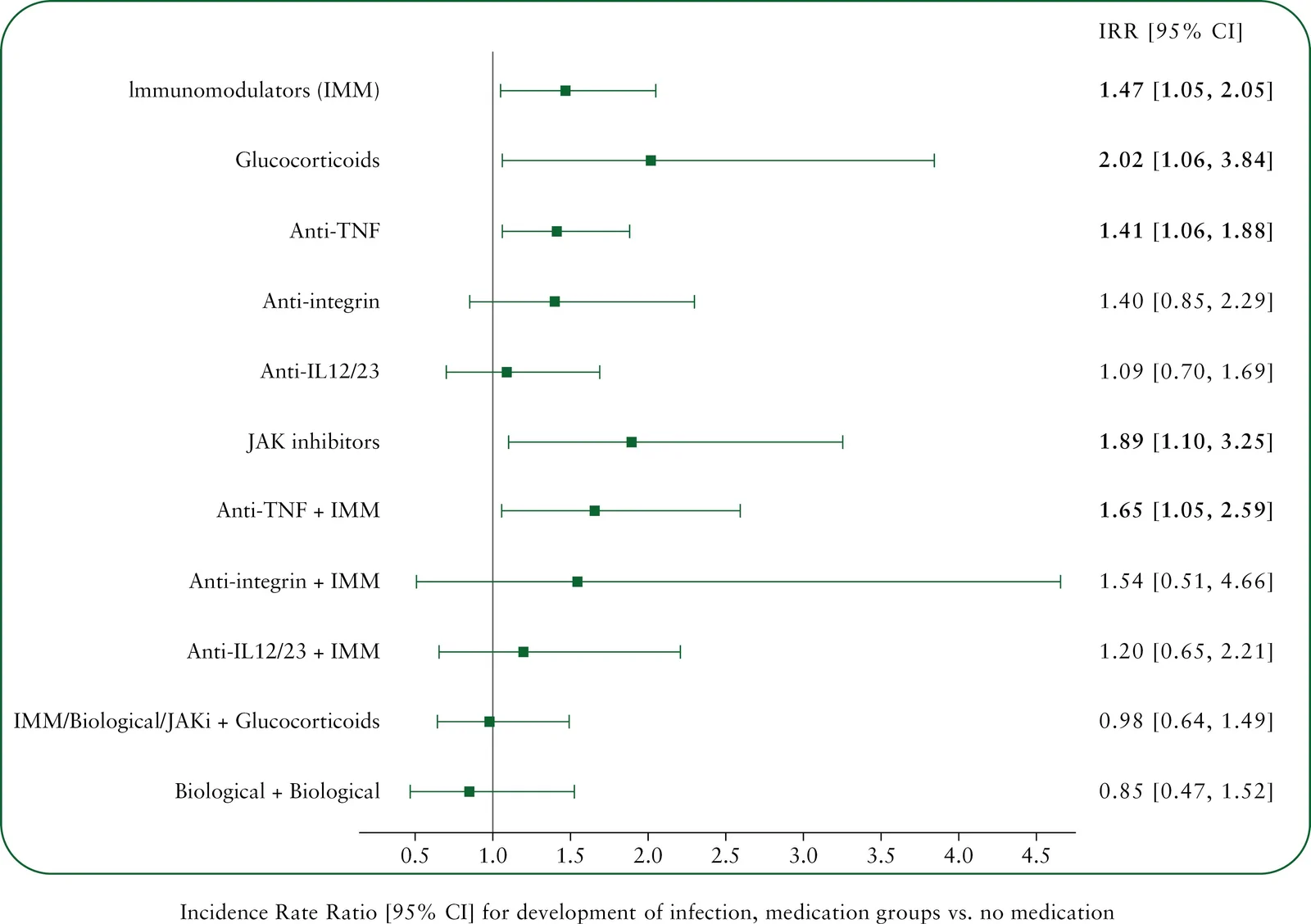
Incidence rate ratios (IRRs) for associations between medication use (ie, medication group vs no medication) and subsequent risk of development of infection in patients with inflammatory bowel disease (IBD) using negative binomial regression. An IRR greater than 1 represents an increase in the risk of developing an infection based on the absence/presence of a predictor. Forest plot only displays medication groups. Model was further adjusted for disease activity, age, sex, disease entity, smoking status, Charlson Comorbidity Index, body mass index, and IBD surgery history. No medication includes mesalamine; immunomodulator (IMM) includes thiopurines (azathioprine, 6-mercaptopurine, 6-thioguanine), tacrolimus, and ciclosporin; glucocorticoids includes prednisone and budesonide; anti-TNF includes infliximab, adalimumab, golimumab, and certolizumab; anti-integrin includes vedolizumab and etrolizumab; anti-IL12/23 includes ustekinumab; JAK inhibitors include tofacitinib, filgotinib, and upadacitinib.

Consistent with the univariable analyses, females (IRR 1.70 [95% CI, 1.35-2.14], *P* < .001) had a higher risk of infections compared to males, and patients with a CCI greater than 2 (IRR 1.69 [95% CI, 1.05-2.74], *P* = .031) also experienced higher infection rates. Furthermore, smoking was associated with increased rates of infections (current vs never IRR 1.43 [95% CI, 1.12-1.81], *P* = .004). In another post-hoc sensitivity analysis replacing CCI with comorbidity domains, only pulmonary comorbidity (IRR 2.17 [95% CI, 1.55-3.02], *P* < .001) was independently associated with increased infection rates, while all other key associations, including female sex, smoking, and most medication effects, remained unchanged. The association for JAK inhibitor use was attenuated but showed a trend toward increased risk (IRR 1.60, *P* = .12) (Supplementary Table 4). Upon excluding UTIs from the multivariable analysis, females still had a statistically significant higher rate of all infections (IRR 1.53 [95% CI, 1.20-1.94], *P* = .01). Importantly, the statistical significance of the other positively associated variables in the initial analysis remained unchanged.

When stratifying for IBD subtype, the increase in the rate of infections was predominantly driven by female sex in both CD and UC, as well as smoking, CCI, and BMI in UC. With regard to medication use, glucocorticoid use (compared to no immunosuppressive medication) was associated with a statistically significant higher rate of infections in UC (IRR 3.10 [95% CI, 1.55-6.18], *P* = .01), but not in CD (Supplementary Table 2 and Supplementary Figure 1).

## 4. Discussion

In this study, we addressed an important knowledge gap concerning mild and moderate infections, which are often overlooked in clinical practice despite their considerable impact on the holistic, personalized management of IBD. Our findings, in a large, multicenter, real-world cohort of IBD patients, indicate an overall infection IR of 172.7 per 100 PY, predominantly characterized by mild (IR 117.5 per 100 PY) and moderate (IR 50.9 per 100 PY) infections. This study uniquely highlighted the impact of various treatment modalities, with the use of immunomodulators, glucocorticoids, anti-TNF agents, and JAK inhibitors being associated with higher infection rates. Additionally, demographic factors such as female sex and higher comorbidity burden were consistently associated with increased infections rates.

To our knowledge, this is the first prospective study that systematically assessed the IRs of infections across various infection categories, with a particular focus on mild and moderate infections, in a real-world cohort of IBD patients. Given the scarcity of similar studies, direct comparisons are challenging. A recent, large-scale retrospective cohort study by Irving et al. in a population-based primary care setting in the United Kingdom, involving 18 289 IBD patients and matched controls, provides a valuable reference point.^18^ Infections during their study period were identified using first-ever diagnostic Read codes covering a range of infection categories defined as “common” infections, similar to those assessed in our study, yet without differentiating between infection severity. They reported an IR of 16.4 per 100 PY for these common infections in UC patients and 17.4 per 100 PY in CD patients, which is substantially (ie, 10-fold) lower than the IRs identified in our study (UC: 158.8 per 100 PY; CD: 184.0 per 100 PY).^18^ This noteworthy discrepancy is most likely attributed to their reliance on diagnostic codes registered by general practitioners, rather than systematic capture of infections using validated measurement tools at fixed intervals, resulting in a probable inaccurate estimation of true IRs. This is further supported by studies reporting IRs of UTIs in primary care in the United Kingdom, where IRs for UTIs approximate the total IR for all infections observed in the study by Irving et al.^26^ As for severe infections, these were also systematically captured in our study. The IR observed for severe infections (4.4 [95% CI, 2.9-6.3] per 100 PY) was comparable, though at the higher end, to those reported in several large postmarketing surveillance studies and safety registries focused on serious infections.^1,5,6,8,27^ This likely reflects differences in cohort size, person-time of exposure, event definitions, as well as the high-resolution data capture used in our study, which may be more sensitive in identifying events of greater clinical concern. In a sensitivity analysis excluding gastrointestinal infections potentially reflecting IBD flares, the IR for severe infections decreased to 3.5 (95% CI, 2.2-5.3) per 100 PY. While this reflects the disproportionate influence of individual events in smaller cohorts, the exclusion did not alter the observed associations in the multivariable model (data not shown).

The most frequently reported infection types in our cohort were URTIs, influenza, and UTIs, which highlights a spectrum that aligns with general population trends. Despite limited studies available for direct comparison, our findings provide nuanced insights on infection dynamics among IBD patients in a real-world setting. For respiratory infections, URTI were consistently among the most reported mild infections in our study, with an IR of 26.9 per 100 PY. Seksik et al. investigated the incidence of benign infections, such as URTI in IBD patients on azathioprine vs patients not on any maintenance therapy. They observed an IR of 2.0 per PY for URTI, with an SD of 2.2, indicating substantial variability.^17^ Notably, the IRs were similar between both groups, suggesting that factors beyond immunosuppression also likely influence infection susceptibility. Interestingly, adults in the general population typically develop around 2 URTIs annually, underscoring the relative frequency of these infections.^28^ The comparatively lower rate for URTI observed in our cohort could be attributed to several factors, including differences in patient management and advances in treatment over the past 2 decades. Additionally, and probably most importantly, the impact of public health measures related to the COVID-19 pandemic—such as social distancing, mask-wearing, and enhanced personal (hand) hygiene, which were particularly emphasized for immunocompromised individuals—undoubtedly contributed to mitigating the transmission of respiratory infections during our study period. Globally, multiple reports highlight that the COVID-19 pandemic significantly impacted the circulation of non-SARS-CoV-2 respiratory viruses, with consistent reductions across multiple geographic regions.^29,30^ This broader trend corroborates our findings, indicating that the observed decrease in respiratory infections and influenza are reflective of a broader global health phenomenon.

The incidence of herpes zoster, while not among the most common infections, is of particular interest due to its clinical relevance and increased risk in immunocompromised populations, including IBD. Our findings on herpes zoster incidence (1.7 [95% CI, 0.9-3.1] per 100 PY) are consistent with other studies in IBD patients, affirming the validity of our data across different epidemiological settings.^31,32^ Additionally, although there are no reports on IR of general UTIs in IBD populations, the IR observed in our study aligns with those reported in primary care settings.^26^ These consistencies suggest that the measures affecting respiratory infection rates did not similarly influence the occurrence of herpes zoster and UTIs, infections which are not typically affected by social distancing protocols.

In examining risk factors for infections in our cohort, univariable analysis revealed statistically significant associations with demographic and clinical factors, including sex, comorbidity burden, and immunosuppressive treatment. Female patients experienced higher rates of infections across all infection severities, with an overall IRR of 1.50 (95% CI, 1.31-1.71, *P* < .001). Intriguingly, even after excluding UTIs from the analysis—an infection predominantly affecting females, with 86% of UTI cases in our study occurring in females—the association, although slightly attenuated, persisted. One could hypothesize about a potential underreporting or differing health-seeking behavior among males. However, this consistent signal suggests an underlying pattern that is not entirely explained by our data or literature, and we currently lack a clear biological explanation for this observation. Regarding the influence of comorbidities on infection risk, patients with a higher comorbidity burden, indicated by higher CCI scores (CCI > 2 vs 0-2), had markedly increased infection rates (IRR 1.69 [95% CI, 1.47-1.94], *P* < .001). This association is supported by data from other studies in IBD patients, which primarily focus on serious infections.^33^ Moreover, it is also consistent with findings from studies involving patients with other immune-mediated inflammatory diseases, such as rheumatoid arthritis, illustrating that a greater comorbidity burden, which also includes older age (ie, > 70 years), inherently increases susceptibility to infections.^34^ A sensitivity analysis using grouped comorbidity domains instead of the CCI confirmed the robustness of this association, with pulmonary comorbidities emerging as the potential primary driver of this signal. This finding highlights the need for clinical awareness of pulmonary conditions as a potential important risk factor for infections in IBD patients. While disease activity was not associated with infection risk in the primary model, a sensitivity analysis in which clinical symptoms were prioritized over FCP in cases of discrepancies revealed a statistically significant association. This finding aligns with clinical expectations, but also underlines the challenges in defining disease activity. Although symptom-based scores like the MIAH are valuable, they may overestimate inflammation in patients with persistent symptoms despite biochemical remission, a group known to comprise up to 40%-50% of patients.^35,36^ In practice, FCP offers a more objective reflection of mucosal inflammation in those cases. Altogether, these findings highlight the importance of contextual interpretation of disease activity when assessing infection risk in real-world settings.

As for the treatment effects observed in the univariable analysis, any form of immunosuppressive treatment was associated with a 49% (IRR 1.49) increase in overall infection rates compared to patients without maintenance therapy. These findings underscored the importance of our subsequent comprehensive multivariable negative binomial regression analysis, accounting for the dynamic nature of medication exposure, with incorporation of washout periods, and disease activity over time.

In the comparative analysis of medication use in our study, we highlighted the differential impact of various treatment modalities on infection risk among IBD patients, after adjusting for demographic factors and other relevant patient characteristics. Immunomodulator monotherapy was associated with a 47% (IRR 1.47) increase in infections compared to no immunosuppressive therapy. Similarly, patients on anti-TNF monotherapy experienced a 41% increase in infection rates, whereas combination therapy with immunomodulators and anti-TNF agents resulted in an increase of 65% in rates of infections. Most strikingly, glucocorticoids more than doubled (IRR 2.02) the risk of infections. While comparative literature on the impact of specific treatments is sparse, our findings align with studies focusing on opportunistic and serious infections, despite differences in outcomes of interest.^2,4,37^

In the study by Irving et al., which focussed on common infections, increased infection rates with biologicals and immunomodulators were also noted, though they did not differentiate between biological types nor account for the dynamic nature of medication use over time, considering only baseline medication use. Interestingly, in their study, the increased risk for glucocorticoids was lower compared to biologicals, despite including vedolizumab and ustekinumab users in that group.^18^ In our study, anti-integrin and anti-IL-12/23 therapies were not associated with an overall increased rate of infections, underscoring their safety profiles in terms of infection risk. These findings align with safety data from RCTs and contribute to an understanding of the benefit-risk profiles, suggesting these treatments may be preferable for patients at higher risk of infections.^38–42^

JAK inhibitors, a more recently introduced class of therapy in the IBD treatment landscape, demonstrated an 89% increase in infection rates, underscoring the substantial risk these agents pose. Data on infections with JAK inhibitors in literature corroborate the increased risk in IBD patients and suggest a dose-dependent increase in infection risk.^43^ Comparative data, however, indicate that the infection risk with JAK inhibitors is similar to that with anti-TNF.^38^ Notably, herpes zoster reactivation is frequent across all JAK inhibitors, prompting recommendations for preventive strategies with prolonged treatment, particularly in high-risk patients, such as older patients and those with higher comorbidity burden.^43,44^

Consistent with the results observed in the univariable analyses, the multivariable analysis further confirmed that female sex and higher comorbidity burden were associated with an increase in infection rates. Additionally, smoking emerged as a risk factor, aligning with extensive literature underscoring the role of smoking in exacerbating infection risk, particularly in immunocompromised populations.^45^ This also reinforces the importance of smoking cessation as part of holistic disease management in IBD. Of note, our analysis revealed some medication combinations not commonly prescribed in standard clinical practice. These combinations, identified due to the nature of our analysis accounting for medication switches and washout periods, have questionable clinical relevance due to the small number of patients involved. Building upon this, when stratifying for CD and UC, clinical characteristics continued to be associated with infection risk. However, the medication signals observed in the overall group were not consistently replicated in these subgroups. This discrepancy is likely attributable to the reduced sample sizes within each medication group after stratification, resulting in a loss of statistical power. Consequently, these findings should be interpreted with caution.

A key strength of our study is its prospective, multicenter design, including patients from both an academic and nonacademic hospital within a real-world setting, which allowed for the collection of detailed data across a substantial real-world patient cohort. The demographic and clinical characteristics of our study participants closely align with those of the population-based IBD South-Limburg cohort, enhancing the generalizability of our findings.^46^ Future inclusion of other regions will help assess potential geographic variations and support nationwide applicability. The use of a validated instrument (ie, the PRIQ^20^) at fixed time intervals ensured accurate data collection, supporting the credibility of infection risk assessments. Additionally, our comprehensive analytical approach, accounting for time-varying covariates and medication washout periods, enabled for nuanced assessments of the impact of various treatment modalities, crucial for informing clinical decision making. Nonetheless, our study has limitations that need to be addressed. The 1-year prospective follow-up coincided with the COVID-19 pandemic, which may have led to an underestimation of overall infection incidence due to reduced respiratory infections from public health measures such as social distancing. However, we showed that this effect did not extend to other infection types, such as UTIs and herpes zoster, suggesting a limited impact on our overall infection incidence findings. Secondly, the real-world nature of our study did not permit precise control over distribution of medication groups. This limitation was particularly evident when stratifying the analysis by IBD subtype, resulting in a possible loss of power to detect whether type of medication impacts infection rates within these subgroups. Thirdly, while severe infections were captured and reported, our study was not powered to evaluate these rare outcomes with high precision given the limited person-time of exposure, and the main strength remains in the systematic assessment of mild and moderate infections. Furthermore, given the heterogeneity in treatment regimens, we did not specifically assess dose-dependent effects, which may limit the granularity of our understanding regarding the association between treatment intensity and infection rate. Lastly, the introduction of newer therapies like S1P-receptor agonists and anti-IL23 blockers after our study period prevented us from assessing their impact on infection risk, making a direction for future research.

In the rapidly evolving landscape of IBD treatment, it becomes apparent that managing IBD extends beyond merely controlling disease activity and fundamentally involves balancing effectiveness with risks. While mild and moderate infections may seem relatively innocuous, their impact on quality of life and potential therapy adherence cannot be underestimated.^13,14^ Recurrent infections, even if not severe, can complicate management of IBD, potentially leading to less beneficial treatment outcomes. Consequently, in our study, it is emphasized that choice of treatment should not only hinge on its potential to control disease activity, but also consider its safety profile, particularly in relation to the individuals infection risk. This is particularly important for specific patient groups, such as (older) individuals with multiple comorbidities and smokers, highlighting the need for personalized treatment strategies.

In conclusion, mild and moderate infections are common in IBD patients. We identified factors that notably elevate infection risks, particularly among women, smokers, and patients with multiple comorbidities. The use of immunomodulators, anti-TNF agents, JAK inhibitors, and corticosteroids further increase these risks, with steroids posing the most important threat. Clinicians should adopt a holistic approach that includes vigilant monitoring, preferably using validated tools, such as the PRIQ, to identify patients with frequent mild or moderate infections. In addition, proactive application of preventive strategies, such as vaccination, optimizing nutritional status, counseling for lifestyle modifications, and careful selection of therapies, should be used to reduce infection risk. Future research could investigate whether recurrent mild or moderate infections are a risk factor for developing severe infections and also focus on the long-term impact of newer therapies and dose-dependent risks associated with current treatments, supported by the planned longer-term follow-up within the PRQI cohort to enable evaluation of rarer outcomes, to better inform clinical decision making and optimize patient outcomes.

## Supplementary Material

jjaf112_Supplementary_Data

## Data Availability

A data management plan for myIBDcoach has been made in collaboration with DataHub MUMC+. DataHub works according to the FAIR principles. Given the use of human research participants in this study, the data contain potentially identifying or sensitive patient information and will therefore not be directly shared. Metadata of myIBDcoach is available for interested researchers and data is available after approval of research proposals by the myIBDcoach content committee (contact: m.pierik@mumc.nl).
