# Supplementary material for: The risk of mild, moderate, and severe infections in inflammatory bowel disease patients—a prospective, multicenter observational cohort study
Source: J Crohns Colitis. 2025 Jul 9;20(11):jjaf112. doi: 10.1093/ecco-jcc/jjaf112 (PMC13760095; doi:10.1093/ecco-jcc/jjaf112)
Supplement: Supplementary file 1 [file jjaf112_supplementary_data.zip › jjaf112_suppl_Supplementary_Tables.docx]

**Supplementary Table 1.** Baseline characteristics of all IBD patients included in the prospective observational cohort study, and stratified by patients developing at least 1 infection during follow-up.

|  | **≥1 infection [n=339]** | **No infection [n=290]** | ***p* value** |
| --- | --- | --- | --- |
| Sex, *n* [%] |  |  | **<0.001** |
| Female | 224 [66.1] | 142 [49.0] |  |
| Male | 115 [33.9] | 148 [51.0] |  |
| IBD type, *n* [%] |  |  | 0.99 |
| CD | 187 [55.2] | 160 [55.2] |  |
| UC | 152 [44.8] | 130 [44.8] |  |
| Age at cohort entry in years, mean [SD] | 49.4 [14.5] | 47.0 [15.1] | **0.04** |
| Disease duration in years^a^, mean [SD] | 12.9 [10.8] | 11.8 [10.8] | 0.21 |
| Montreal A diagnosis^b^, *n* [%] |  |  | 0.76 |
| A1: <17 | 18 [5.3] | 12 [4.1] |  |
| A2: 17-40 | 194 [57.2] | 171 [59.0] |  |
| A3: ≥ 40 | 127 [37.5] | 107 [36.9] |  |
| Disease location UC^c^, *n* [%] |  |  | 0.57 |
| E1: ulcerative proctitis | 32 [21.1] | 21 [16.2] |  |
| E2: left-sided | 66 [43.4] | 59 [45.4] |  |
| E3: extensive | 54 [35.5] | 50 [38.5] |  |
| Disease location CD^c^, *n* [%] |  |  | 0.53 |
| L1: ileal | 64 [34.2] | 49 [30.6] |  |
| L2: colonic | 50 [26.7] | 39 [24.4] |  |
| L3: ileocolonic | 73 [39.0] | 72 [45.0] |  |
| P: Perianal involvement | 39 [20.9] | 36 [22.5] | 0.71 |
| U: Upper disease | 6 [3.2] | 10 [6.3] | 0.18 |
| Disease behaviour^c^, *n* [%] |  |  | 0.64 |
| B1: inflammatory | 144 [77.0] | 117 [73.1] |  |
| B2: stricturing | 29 [15.5] | 27 [16.9] |  |
| B3: penetrating | 14 [7.5] | 16 [10.0] |  |
| IBD surgery resection in history, *n* [%] | 78 [23.0] | 59 [20.3] | 0.42 |
| Stoma/pouch, *n* [%] | 15 [4.4] | 6 [2.1] | 0.23 |
| IBD in family^d^ | 94 [27.7] | 90 [31.0] | 0.65 |
| Smoking status, *n* [%] |  |  | 0.06 |
| Never | 133 [39.2] | 141 [48.6] |  |
| Ever smoked | 164 [48.4] | 117 [40.3] |  |
| Currently smoking | 42 [12.4] | 32 [11.0] |  |
| CCI (Charlson Comorbidity Index), *n* [%] |  |  | **0.005** |
| 0 | 136 [40.1] | 142 [49.0] |  |
| 1-2 | 129 [38.1] | 112 [38.6] |  |
| >2 | 74 [21.8] | 36 [12.4] |  |
| BMI in kg/m^2^, mean [SD] | 26.7 [5.1] | 25.8 [4.1] | **0.008** |
| BMI category^e^, *n* [%] |  |  | 0.34 |
| Underweight | 5 [1.5] | 6 [2.1] |  |
| Normal weight | 138 [40.7] | 132 [45.5] |  |
| Overweight | 125 [36.9] | 106 [36.6] |  |
| Obesity | 71 [20.9] | 46 [15.9] |  |
| History of serious infection, *n* [%] |  |  | 0.25 |
| Tuberculosis | 3 [0.9] | 4 [1.4] |  |
| Hepatitis B | 2 [0.6] | 1 [0.3] |  |
| HIV | 0 [0.0] | 0 [0.0] |  |
| Comorbidities, n (%) |  |  |  |
| Heart disease | 29 [8.6] | 12 [4.1] | **0.03** |
| Hypertension | 53 [15.6] | 26 [9.0] | **0.01** |
| Lung disease | 35 10.3] | 17 [5.9] | **0.04** |
| Intermittent claudication | 13 [3.8] | 8 [2.8] | 0.45 |
| Diabetes Mellitus | 21 [6.2] | 7 [2.4] | **0.02** |
| Kidney disease | 5 [1.5] | 5 [1.7] | 0.80 |
| Neurological disease | 4 [1.2] | 5 [1.7] | 0.57 |
| Liver disease | 3 [0.9] | 1 [0.3] | 0.40 |
| Cancer | 9 [2.7] | 8 [2.8] | 0.93 |
| Depression | 20 [5.9] | 14 [4.8] | 0.55 |
| Hypercholesterolemia | 29 [8.6] | 12 [4.1] | **0.03** |
| Stroke problems | 5 [1.5] | 0 [0.0] | **0.04** |
| Osteoarthritis | 8 [2.4] | 1 [0.3] | **0.03** |
| Back pain | 60 [17.7] | 34 [11.7] | **0.04** |
| Rheumatoid arthritis | 32 [9.4] | 14 [4.8] | **0.03** |
| Ankylosing spondylitis | 21 [6.2] | 6 [2.1] | **0.01** |
| Primary Sclerosing cholangitis | 3 [0.9] | 1 [0.3] | 0.40 |
| Psoriasis | 21 [6.2] | 27 [9.3] | 0.14 |
| Baseline medication use^f^, *n* [%] |  |  | 0.287 |
| No medication | 110 [32.5] | 103 [35.5] |  |
| Immunomodulators (IMM) | 47 [13.9] | 41 [14.1] |  |
| Glucocorticoids | 13 [3.8] | 11 [3.8] |  |
| Anti-TNF | 81 [23.9] | 57 [19.7] |  |
| Anti-integrin | 18 [5.3] | 13 [4.5] |  |
| Anti-IL12/23 | 14 [4.1] | 15 [5.2] |  |
| JAK inhibitors | 4 [1.2] | 2 [0.7] |  |
| Biological + IMM | 34 [10.0] | 19 [6.6] |  |
| Biological/IMM/SM + Glucocorticoids | 17 [5.0] | 24 [8.3] |  |
| Biological + Biological | 1 [0.3] | 5 [1.7] |  |

IBD, Inflammatory bowel disease; CD, Crohn’s disease; UC, ulcerative colitis; *n*, number of patients; SD, standard deviation; BMI; body mass index

^¥^ Ulcerative colitis including indeterminate colitis. In total 5 cases. ^a^ Disease duration at time of cohort entry in years. ^b^ Age at time of diagnosis according to Montreal Classification. ^c^ Current disease phenotype according to Montreal Classification. ^d^ IBD in family status not known in *n*=57 (*n*=34 CD, *n*=38 UC) patients ^e^ underweight <18.50 kg/m2, normal weight 18.50 – 24.90 kg/m2, overweight 25.0 – 29.90 kg/m2 and Obesity ≥30.0 kg/m2. ^f^ Medication at time of cohort entry. No medication includes mesalamine; IMM includes thiopurines (azathioprine, 6-mercaptopurine, 6-thioguanine), tacrolimus and ciclosporin; Glucocorticoids includes prednisone and budesonide; Anti-TNF includes infliximab, adalimumab, golimumab, and certolizumab; Anti-integrin includes vedolizumab and etrolizumab; Anti-IL12/23 includes Ustekinumab; JAK inhibitors include Tofacitinib, Filgotinib and Upadacitinib.

**Supplementary Table 2.** Incidence rate ratios for associations between predictors and subsequent risk of development of infection using negative binomial regression stratified for IBD type.

|  | **All Infections†** | | | |
| --- | --- | --- | --- | --- |
|  | **CD** | | **UC** | |
|  | **IRR [95% CI]** | ***p-*value** | **IRR [95% CI]** | ***p-*value** |
| Medication groups |  |  |  |  |
| No medication | REF |  | REF |  |
| Immunomodulators (IMM) | 1.49 [0.92-2.43] | 0.11 | 1.23 [0.82-1.83] | 0.32 |
| Glucocorticoids | 1.37 [0.51-3.70] | 0.53 | **3.10 [1.55-6.18]** | **0.01*** |
| Anti-TNF | 1.29 [0.88-1.89] | 0.20 | 1.40 [0.90-2.19] | 0.14 |
| Anti-integrin | 0.80 [0.37-1.72] | 0.56 | **2.15 [1.18-3.91]** | **0.012*** |
| Anti-IL12/23 | 0.98 [0.58-1.65] | 0.94 | 0.98 [0.41-2.31] | 0.96 |
| JAK inhibitors | 1.62 [0.63-4.19] | 0.32 | 2.24 [0.92-5.45] | 0.076 |
| Anti-TNF + IMM | 1.80 [0.99-3.28] | 0.05 | 1.42 [0.73-2.74] | 0.30 |
| Anti-integrin + IMM | NA |  | 2.02 [0.55-7.44] | 0.29 |
| Anti-IL12/23 + IMM | 1.20 [0.64-2.26] | 0.58 | NA |  |
| IMM/Biological/JAKi + Glucocorticoids | 0.70 [0.38-1.27] | 0.24 | 1.25 [0.69-2.28] | 0.47 |
| Biological + Biological | 0.85 [0.43-1.69] | 0.65 | 0.70 [0.39-1.27] | 0.24 |
| Age at cohort entry | 1.01 [0.99-1.03] | 0.40 | 0.99 [0.97-1.00] | 0.13 |
| Sex, female | **1.72 [1.24-2.39]** | **0.001*** | **1.80 [1.34-2.40]** | **<0.001*** |
| Disease activity, yes | 0.97 [0.74-1.26] | 0.80 | 0.87 [0.64-1.17] | 0.36 |
| Smoking |  |  |  |  |
| Never | REF |  | REF |  |
| Former | 1.29 [0.86-1.94] | 0.21 | 1.43 [0.67-3.09] | 0.36 |
| Current | 1.31 [0.94-1.83] | 0.12 | **1.48 [1.07-2.05]** | **0.017*** |
| Charlson comorbidity index |  |  |  |  |
| 0 | REF |  | REF |  |
| 1-2 | 0.76 [0.46-1.27] | 0.30 | **1.75 [1.11-2.77]** | **0.017*** |
| >2 | 1.26 [0.61-2.59] | 0.53 | **3.31 [1.92-5.71]** | **<0.001*** |
| BMI category |  |  |  |  |
| Normal weight | REF |  | REF |  |
| Underweight | 1.13 [0.30-4.20] | 0.86 | 1.63 [0.67-3.96] | 0.28 |
| Overweight | 0.78 [0.55-1.08] | 0.13 | **1.61 [1.16-2.23]** | **0.004*** |
| Obesity | 1.32 [0.87-2.00] | 0.19 | 1.17 [0.78-1.75] | 0.44 |
| IBD surgery resection history, yes | 1.03 [0.74-1.42] | 0.88 | 1.36 [0.77-2.40] | 0.29 |

IRR, Incidence rate ratio; CI, confidence interval; REF, reference category; UC, ulcerative colitis; BMI, Body Mass Index. * Significant p value <0.05.

A IRR greater than 1 represents an increase in the risk of developing an infection based on the absence/presence (categorical) or increase in 1 unit value (continuous) of an predictor. Model includes both time-varying predictors (i.e. medication groups, disease activity) and time-invariant predictors (i.e. value at cohort entry: age, sex, smoking status, Charlson comorbidity index, BMI, IBD surgery history)

No medication includes mesalamine; IMM includes thiopurines (azathioprine, 6-mercaptopurine, 6-thioguanine), tacrolimus and ciclosporin; Glucocorticoids includes prednisone and budesonide; Anti-TNF includes infliximab, adalimumab, golimumab, and certolizumab; Anti-integrin includes vedolizumab and etrolizumab; Anti-IL12/23 includes Ustekinumab; JAK inhibitors include Tofacitinib, Filgotinib and Upadacitinib.

BMI: underweight <18.50 kg/m2, normal weight 18.50 – 24.90 kg/m2, overweight 25.0 – 29.90 kg/m2 and Obesity ≥30.0 kg/m2

**Supplementary Table 3.** Incidence rate ratios for associations between predictors and subsequent risk of development of infection in patients with IBD using negative binomial regression **- Sensitivity analysis disease activity.**

|  | **All Infections†** | |
| --- | --- | --- |
|  | **Incidence rate ratio [95% CI]** | ***p-*value** |
| Medication groups |  |  |
| No medication | REF |  |
| Immunomodulators (IMM) | **1.49 [1.07-2.08]** | **0.019*** |
| Glucocorticoids | **1.93 [1.01-3.66]** | **0.046*** |
| Anti-TNF | **1.41 [1.06-1.86]** | **0.018*** |
| Anti-integrin | 1.42 [0.87-2.32] | 0.17 |
| Anti-IL12/23 | 1.04 [0.67-1.61] | 0.86 |
| JAK inhibitors | **1.77 [0.92-3.40]** | **0.045*** |
| Anti-TNF + IMM | **1.59 [1.03-2.47]** | **0.038*** |
| Anti-integrin + IMM | 1.59 [0.51-4.96] | 0.43 |
| Anti-IL12/23 + IMM | 1.02 [0.55-1.87] | 0.95 |
| IMM/Biological/JAKi + Glucocorticoids | 0.93 [0.61-1.43] | 0.75 |
| Biological + Biological | 0.79 [0.43-1.46] | 0.46 |
| Age at cohort entry | 1.00 [0.99-1.02] | 0.72 |
| Sex, female | **1.69 [1.35-2.12]** | **<0.001*** |
| Disease activity, yes | **1.30 [1.07-1.59]** | **0.009*** |
| Disease entity, UC | 0.93 [0.73-1.19] | 0.58 |
| Smoking |  |  |
| Never | REF |  |
| Former | 1.31 [0.91-1.88] | 0.15 |
| Current | **1.42 [1.12-1.80]** | **0.004*** |
| Charlson comorbidity index |  |  |
| 0 | REF |  |
| 1-2 | 0.98 [0.69-1.39] | 0.91 |
| >2 | **1.64 [1.02-2.64]** | **0.042*** |
| BMI category |  |  |
| Normal weight | REF |  |
| Underweight | 1.21 [0.42-3.51] | 0.72 |
| Overweight | 1.05 [0.83-1.34] | 0.68 |
| Obesity | 1.27 [0.94-1.71] | 0.12 |
| IBD surgery resection history, yes | 1.00 [0.75-1.33] | 0.98 |

IRR, Incidence rate ratio; CI, confidence interval; REF, reference category; UC, ulcerative colitis; BMI, Body Mass Index. * Significant p value <0.05.

A IRR greater than 1 represents an increase in the risk of developing an infection based on the absence/presence (categorical) or increase in 1 unit value (continuous) of an predictor. GEE model includes both time-varying predictors (i.e. medication groups, disease activity) and time-invariant predictors (i.e. value at cohort entry: age, sex, disease entity, smoking status, Charlson comorbidity index, BMI, IBD surgery history)

No medication includes mesalamine; IMM includes thiopurines (azathioprine, 6-mercaptopurine, 6-thioguanine), tacrolimus and ciclosporin; Glucocorticoids includes prednisone and budesonide; Anti-TNF includes infliximab, adalimumab, golimumab, and certolizumab; Anti-integrin includes vedolizumab and etrolizumab; Anti-IL12/23 includes Ustekinumab; JAK inhibitors include Tofacitinib, Filgotinib and Upadacitinib.

BMI: underweight <18.50 kg/m2, normal weight 18.50 – 24.90 kg/m2, overweight 25.0 – 29.90 kg/m2 and Obesity ≥30.0 kg/m2

**Supplementary Table 4.** Incidence rate ratios for associations between predictors and subsequent risk of development of infection in patients with IBD using negative binomial regression – **comorbidity sensitivity analysis.**

|  | **All Infections†** | |
| --- | --- | --- |
|  | **Incidence rate ratio [95% CI]** | ***p-*value** |
| Medication groups |  |  |
| No medication | REF |  |
| Immunomodulators (IMM) | **1.36 [0.99–1.87]** | **0.045*** |
| Glucocorticoids | **1.86 [1.06–3.25]** | **0.030*** |
| Anti-TNF | **1.35 [1.03–1.77]** | **0.027*** |
| Anti-integrin | 1.27 [0.78–2.08] | 0.33 |
| Anti-IL12/23 | 0.93 [0.60–1.44] | 0.74 |
| JAK inhibitors | 1.60 [0.88–2.94] | 0.13 |
| Anti-TNF + IMM | **1.76 [1.11–2.79]** | **0.016*** |
| Anti-integrin + IMM | 1.48 [0.55–3.99] | 0.44 |
| Anti-IL12/23 + IMM | 1.21 [0.64–2.28] | 0.56 |
| IMM/Biological/JAKi + Glucocorticoids | 0.97 [0.64–1.48] | 0.89 |
| Biological + Biological | 0.77 [0.34–1.77] | 0.54 |
| Age at cohort entry | 1.01 [0.99-1.02] | 0.10 |
| Sex, female | **1.55 [1.24-1.93]** | **<0.001*** |
| Disease activity, yes | 0.95 [0.78-1.20] | 0.65 |
| Disease entity, UC | 0.91 [0.72-1.15] | 0.67 |
| Smoking |  |  |
| Never | REF |  |
| Former | 1.38 [0.95-2.01] | 0.091 |
| Current | **1.36 [1.07-1.73]** | **0.004*** |
| Comorbidities |  |  |
| Cardiovascular | 1.15 [0.89–1.49] | 0.30 |
| Metabolic | 1.10 [0.68–1.78] | 0.70 |
| Pulmonary | **2.17 [1.55–3.02]** | **<0.001*** |
| Renal | 1.02 [0.42–2.51] | 0.96 |
| Hepatic | 0.82 [0.30–2.24] | 0.69 |
| Neuro/Psych | 1.09 [0.74–1.61] | 0.66 |
| Musculoskeletal | 1.23 [0.94–1.62] | 0.13 |
| IMID | 1.19 [0.91–1.54] | 0.20 |
| Malignancy | 0.63 [0.34–1.20] | 0.16 |
| BMI category |  |  |
| Normal weight | REF |  |
| Underweight | 1.14 [0.38-3.41] | 0.82 |
| Overweight | 1.02 [0.80-1.29] | 0.88 |
| Obesity | 1.27 [0.94-1.70] | 0.12 |
| IBD surgery resection history, yes | 0.96 [0.73-1.27] | 0.77 |

IRR, Incidence rate ratio; CI, confidence interval; REF, reference category; UC, ulcerative colitis; BMI, Body Mass Index. * Significant p value <0.05.

A IRR greater than 1 represents an increase in the risk of developing an infection based on the absence/presence (categorical) or increase in 1 unit value (continuous) of an predictor. GEE model includes both time-varying predictors (i.e. medication groups, disease activity) and time-invariant predictors (i.e. value at cohort entry: age, sex, disease entity, smoking status, comorbidities, BMI, IBD surgery history)

No medication includes mesalamine; IMM includes thiopurines (azathioprine, 6-mercaptopurine, 6-thioguanine), tacrolimus and ciclosporin; Glucocorticoids includes prednisone and budesonide; Anti-TNF includes infliximab, adalimumab, golimumab, and certolizumab; Anti-integrin includes vedolizumab and etrolizumab; Anti-IL12/23 includes Ustekinumab; JAK inhibitors include Tofacitinib, Filgotinib and Upadacitinib.

BMI: underweight <18.50 kg/m2, normal weight 18.50 – 24.90 kg/m2, overweight 25.0 – 29.90 kg/m2 and Obesity ≥30.0 kg/m2
