# Supplementary material for: The risk of mild, moderate, and severe infections in inflammatory bowel disease patients—a prospective, multicenter observational cohort study
Source: J Crohns Colitis. 2025 Jul 9;20(11):jjaf112. doi: 10.1093/ecco-jcc/jjaf112 (PMC13760095; doi:10.1093/ecco-jcc/jjaf112)
Supplement: Supplementary file 1 [file jjaf112_supplementary_data.zip › SuppFigure1_ForestPlotCDvsUC.pdf]

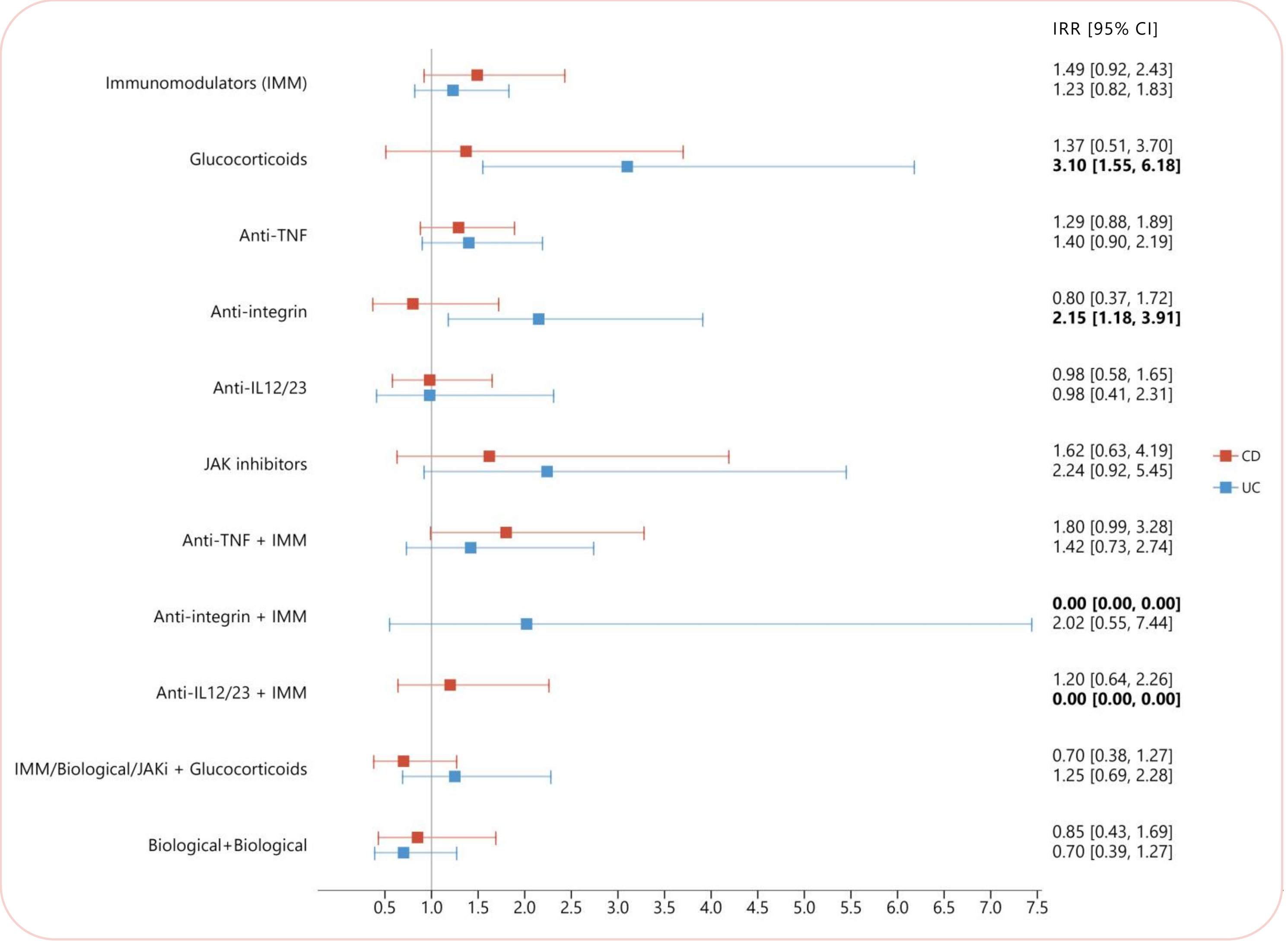

Incidence Rate Ratio [95% CI] for development of infection stratified for IBD type, medication groups vs. no medication
