# Supplementary material for: The risk of mild, moderate, and severe infections in inflammatory bowel disease patients—a prospective, multicenter observational cohort study
Source: J Crohns Colitis. 2025 Jul 9;20(11):jjaf112. doi: 10.1093/ecco-jcc/jjaf112 (PMC13760095; doi:10.1093/ecco-jcc/jjaf112)
Supplement: Supplementary file 1 [file jjaf112_supplementary_data.zip › Supplementary figure 1.docx]

**Supplementary figure 1.** Incidence rate ratios [IRR] for associations between medication use (*i.e*. medication group versus no medication) and subsequent risk of development of infection in patients with IBD using negative binomial regression, stratified for IBD type. An IRR greater than 1 represents an increase in the risk of developing an infection based on the absence/presence of an predictor. Forest plot only displays medication groups. Model was further adjusted for disease activity, age, sex, disease entity, smoking status, Charlson comorbidity index, BMI, and IBD surgery history.

No medication includes mesalamine; IMM includes thiopurines (azathioprine, 6-mercaptopurine, 6-thioguanine), tacrolimus and ciclosporin; Glucocorticoids includes prednisone and budesonide; Anti-TNF includes infliximab, adalimumab, golimumab, and certolizumab; Anti-integrin includes vedolizumab and etrolizumab; Anti-IL12/23 includes Ustekinumab; JAK inhibitors include Tofacitinib, Filgotinib and Upadacitinib

*The instances with IRR of 0.00 reflects that no patients were on that combination of medications
